# Drug-induced hypesthesia due to dexamethasone: A case report

**DOI:** 10.1097/MD.0000000000041990

**Published:** 2025-05-09

**Authors:** WenHao Jia, Bo Yang

**Affiliations:** a Department of Pharmacy, Zhejiang Academy of Traditional Chinese Medicine, Tongde Hospital of Zhejiang Province, Hangzhou, PR China.

**Keywords:** adverse drug reactions, case report, dexamethasone, hypesthesia

## Abstract

**Rationale:**

Systemic glucocorticoids are widely used in the treatment of various inflammatory and autoimmune conditions. This report describes the first known case of drug-induced hypesthesia following dexamethasone administration in a patient without prior neurological conditions.

**Patient concerns:**

A 38-year-old female patient presented with bothersome and intolerable tinnitus and was diagnosed with sudden sensorineural hearing loss. She was treated with dexamethasone (10 mg IVGTT once daily) for 6 days. On the 5th day of treatment, the patient began experiencing fatigue, followed by decreased sensitivity to pain, temperature, and touch, anhidrosis, and lack of satiety.

**Diagnoses:**

Drug-induced hypesthesia.

**Interventions:**

Dexamethasone was discontinued after completing the 6-day treatment course. The patient was then prescribed omeprazole and mecobalamin for supportive care.

**Outcomes:**

The patient’s sensory impairment began to resolve 3 days after stopping dexamethasone. Follow-up 1 week later confirmed complete recovery without recurrence of hypesthesia.

**Lessons:**

This case highlights the importance of monitoring patients for hypesthesia during dexamethasone therapy, even in the short term. Clinicians should be aware of this potential adverse effect and consider alternative treatments if necessary. Mecobalamin may be a supportive option for managing hypesthesia in such cases.

## 1. Introduction

Hypoesthesia refers to a reduction or weakening in the ability to perceive pain, temperature, touch, or vibration. This condition implies that the senses can still detect stimuli, albeit with potentially diminished or incomplete responses. Hypoesthesia is commonly associated with diseases of the central or peripheral nervous system. However, it can also be influenced by alcohol, toxins, or specific medications. To date, there have been no reports of hypesthesia induced by dexamethasone. We present a case report of a patient with sudden deafness who developed reversible hypesthesia following treatment with dexamethasone, aiming to raise clinical awareness regarding such adverse reactions.

## 2. Case presentation

A 38-year-old female patient presented to our hospital with bothersome and intolerable tinnitus. The patient had previously been in good health and denied any history of chronic diseases. Physical examination revealed no significant abnormalities in the tympanic membranes. Pure-tone audiometry indicated an elevated hearing threshold at 250 Hz in the left ear. Distortion product otoacoustic emissions testing showed no responses at 4000 and 8000 Hz in the right ear. Three-dimensional tympanometry examinations revealed type A curves bilaterally. Based on the patient’s clinical presentation and ancillary test results, a preliminary diagnosis of sudden sensorineural hearing loss was made. Treatment with dexamethasone 10 mg via intravenous infusion once daily was prescribed for outpatient infusion therapy. Initial treatment will be for 3 days, with the possibility of extending treatment for another 3 days if there is clinical improvement.

**Figure 1.**
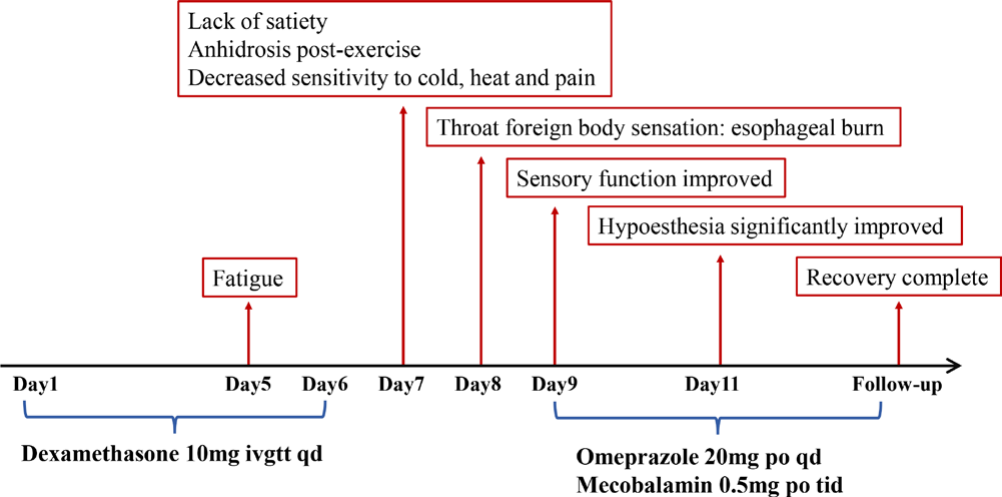
The brief summary of the patient’s history.

During the initial 3 days of treatment, the patient reported that her tinnitus symptoms had significantly reduced, she felt well, and experienced no obvious discomfort. However, from the afternoon of the 5th day of drug administration, the patient began feeling fatigued, which continued through the 6th day evening. At the time, this was attributed to work-related stress, and the symptoms alleviated after adequate rest, so no further attention was given. On the 2nd day after discontinuing the medication (i.e., the 7th day from the start of treatment), the patient frequently felt hungry. Despite eating more than usual during meals, she did not feel satiated. Additionally, after performing moderate-intensity fitness exercise for 1 hour, the patient did not sweat as usual. During bathing, the patient noticed a decreased sensitivity to changes in water temperature. On the 8th day, the patient went to work as usual. In the evening, upon returning home, she felt a sensation of a foreign body in her throat, realizing that she might have been scalded by hot soup while eating noodles at lunchtime. Simultaneously, when pricking her limbs with a sharp object, she felt less sensitive to pain. On the 9th day, the patient visited the hospital. Physical examination revealed negative findings for cranial nerves, and limb strength was graded V in all 4 extremities. Head MRI, including diffusion-weighted imaging and magnetic resonance angiography, showed no significant abnormalities. Electromyography and evoked potential tests were also unremarkable. Blood biochemistry, including electrolyte levels, showed no abnormalities. While deliberating on subsequent treatment options, the patient reported in the afternoon that she could finally feel the coldness of the water while washing her hands, which was considered a sign of improved sensory function. The patient was prescribed omeprazole enteric-coated tablets (20 mg po qd) and mecobalamin tablets (0.5 mg po tid) and advised to observe at home temporarily. On the eleventh day, the patient reported that her sensory impairment had largely resolved. Follow-up 1 week later showed complete recovery. The clinical process and treatment schedule of the patient are shown in Figure 1.

## 3. Discussion

Steroid corticosteroids are essential for the treatment of many inflammatory, allergic, immunological, and neoplastic conditions. However, their systemic application is also a common cause of iatrogenic disorders.^[1]^ Previous reports have involved multiple organ systems, including the skin, eyes, cardiovascular system, gastrointestinal tract, metabolism, bones, nervous system, and psychiatric health.^[2]^ Regarding adverse effects on the nervous system, reports have primarily focused on psychiatric and cognitive symptom changes.^[3,4]^ To our knowledge, there have been no reports of hypesthesia caused by corticosteroids.

A detailed medical and medication history was obtained from the patient, who denied other illnesses and reported no recent use of other medications or supplements. In this case, the patient exhibited a series of adverse reactions, including somnolence, anhidrosis postexercise, lack of satiety, and decreased sensitivity to cold, heat, and pain. Apart from somnolence, which may be related to the involvement of the central nervous system, the other reactions fall under the category of hypesthesia. Specifically, anhidrosis refers to the inability to sweat normally during exercise or other situations, which may be related to thermoregulatory dysfunction. Lack of satiety refers to the inability to perceive a state of fullness, which may be related to impaired sensory feedback mechanisms in the gastrointestinal tract. Decreased sensitivity to cold, heat and pain refers to hypesthesia, characterized by reduced perception of external temperature changes and pain stimuli, usually involving the dysfunction of sensory nerve fibers in the skin and mucous membranes. These symptoms have a clear temporal association with the administration of dexamethasone. The Naranjo method,^[5]^ an algorithm used to assess the likelihood of adverse drug reactions, indicated that dexamethasone was likely responsible for the hypesthesia, yielding a probability score of + 5 (Table 1).

**Table 1:** Naranjo’s scale for the likelihood of hypoesthesia caused by dexamethasone.

Naranjo’s scale				
Question	Yes	No	Don’t know	Score for ADR
1. Are there previous conclusive reports on this reaction?	+1	0	0	0
2. Did the adverse event appear after the suspected drug was administered?	+2	‐1	0	+2
3. Did the adverse reaction improve when the drug was discontinued or a specific antagonist was administered?	+1	0	0	+1
4. Did the adverse reaction reappear when the drug was readministered?	+2	‐1	0	0
5. Are there alternate causes (other than the drug) that could have solely caused the reaction?	-1	+2	0	+2
6. Did the reaction reappear when a placebo was administered?	‐1	+1	0	0
7. Was the drug detected in the blood (or other fluids) at a concentration known to be toxic?	+1	0	0	0
8. Was the reaction more severe when the dose was increased or less severe when the dose was decreased?	+1	0	0	0
9. Did the patient have a similar reaction to the same or similar drugs in any previous exposure?	+1	0	0	0
10. Was the adverse event confirmed by objective evidence?	+1	0	0	0
Total				+5

Scoring for Naranjo’s algorithm: ≥9 = definite; 5–8 = probable; 1–4 = possible; 0 = doubtful.

Given the absence of alternative causes and the temporal relationship between dexamethasone administration and the onset of hypesthesia, it is reasonable to hypothesize that dexamethasone may have contributed to these symptoms. The pathophysiological mechanism behind dexamethasone-induced hypesthesia is not fully understood but could involve direct neurotoxicity, alterations in neurotransmitter function, or disruption of the blood–brain barrier.^[6,7]^ In this case, the patient’s symptoms of sensory impairment suggest a possible widespread neurological issue, particularly affecting the peripheral nervous system.

Although hypesthesia may result from various underlying diseases, it is essential to further evaluate and explore these possibilities. Symptoms of some conditions may persist for a period even after the cessation of triggering factors, exhibiting characteristics of delayed recovery. For example, in neurological diseases such as multiple sclerosis or multifocal motor neuropathy, while MRI results in this case showed no abnormalities, these diseases cannot be completely ruled out, particularly in their early stages.^[8]^ Further neurophysiological examinations or long-term follow-up could help exclude these diagnoses. Additionally, metabolic or nutritional deficiencies, including vitamin B12, folate, or other micronutrients, can lead to peripheral neuropathy, which in turn causes sensory abnormalities.^[9]^ Therefore, it is advisable to assess patients’ nutritional status more thoroughly, including measuring serum vitamin levels, in similar cases moving forward. Autoimmune diseases like Guillain–Barré syndrome or other autoimmune-related peripheral neuropathies may also present with similar symptoms.^[10]^ Although these conditions are often accompanied by noticeable limb weakness, distinguishing them based solely on sensory symptoms can be challenging in the early stages. Thus, for unexplained sensory disorders, considering further immunological testing is necessary.

In summary, hypesthesia is a complex neurological symptom with diverse etiologies, including neural, metabolic, infectious, and traumatic factors. Additionally, during pharmacotherapy, numerous medications can also trigger this side effect, making the identification of potential risk drugs crucial for both clinicians and patients. For example, certain antidepressants such as selective serotonin reuptake inhibitors can indirectly affect sensory processing by altering neurotransmitter levels in the brain.^[11]^ Local anesthetics like lidocaine achieve their effect by blocking the conduction of nerve impulses.^[12]^ Antiarrhythmic drugs such as amiodarone may cause hypesthesia through mechanisms involving direct neurotoxicity, affecting metabolic and nutritional states.^[13]^ Chemotherapeutic agents sometimes damage peripheral nerves, resulting in sensory abnormalities.^[14]^ Some antibiotics^[15]^ and anticonvulsants such as phenytoin,^[16]^ although less commonly, can still impact the function of sensory nerves. These drugs disrupt normal nervous system function through various mechanisms, ultimately causing hypesthesia.

Management of such adverse reactions is critical to prevent further complications. Literature review shows that, apart from conditions such as cataracts, accelerated atherosclerotic vascular disease, and skeletal effects (such as osteoporosis and osteonecrosis), most glucocorticoid toxicities can gradually show at least partial reversal after discontinuation of the medication.^[17]^ Therefore, upon identification of adverse reactions like hypesthesia, immediate cessation of the drug should be prioritized. However, in appropriate cases, switching to different corticosteroids (such as methylprednisolone, which has fewer adverse effects) may be a viable alternative.^[18]^ Additionally, close monitoring of the patient’s response and regular reevaluation are necessary to ensure the best possible outcome while minimizing the risk of iatrogenic complications. In this case, the patient’s symptoms of hypesthesia improved following the discontinuation of dexamethasone, providing empirical evidence that supports the effectiveness of prompt management strategies.

This case has some limitations. First, this is a single-case study, which limits the generalizability of the conclusions. Second, we did not readminister dexamethasone to strengthen the validation of the causal relationship between the drug and the adverse reaction. Third, we were unable to determine whether the application of mecobalamin had any beneficial effect on the alleviation of symptoms. Further research will help to better understand the incidence and mechanisms of dexamethasone-induced hypesthesia and to develop more effective prevention and management strategies.

## 4. Conclusion

In summary, we report a case of hypesthesia induced by dexamethasone. This case serves as a reminder of the necessity for close monitoring during dexamethasone therapy. Although rare, healthcare providers should be aware of such potential adverse reactions. This case can provide empirical reference for the early recognition and management of hypesthesia induced by glucocorticoids in clinical practice.

## Author contributions

**Conceptualization:** Bo Yang.

**Data curation:** Bo Yang.

**Resources:** WenHao Jia.

**Writing – original draft:** WenHao Jia.

**Writing – review & editing:** Bo Yang.
